# White muscle disease in foals: focus on selenium soil content. A case series

**DOI:** 10.1186/s12917-017-1040-5

**Published:** 2017-05-03

**Authors:** Catherine Delesalle, Marco de Bruijn, Sanne Wilmink, Hilde Vandendriessche, Gerben Mol, Berit Boshuizen, Lukas Plancke, Guy Grinwis

**Affiliations:** 1 Department of Comparative Physiology and Biometrics, Faculty of Veterinary Medicine, Ghent University, Ghent, Belgium; 2 Wolvega Equine Hospital, Weststellingwerf, The Netherlands; 3 Department of Clinical Veterinary Science, University of Bristol, Langford House, Langford, North Somerset UK; 4 Division of Crop Biotechnics, KU Leuven and Soil Service of Belgium, Leuven, Belgium; 5 Alterra, Wageningen University & Research Centre, Wageningen, The Netherlands; 6 Department of Pathobiology, Faculty of Veterinary Medicine, Utrecht University, Utrecht, The Netherlands

**Keywords:** Selenium, Vitamin E, Liver disease, Nutritional myopathy, Soil analysis

## Abstract

**Background:**

White muscle disease (WMD) is a nutritional myopathy caused by selenium (Se) deficiency. In most soils, Se is present in low concentrations, sometimes even below 0.2 mg/kg, a trend which is seen in many countries. Apart from total soil Se concentrations, soil conditions may be such that the bio-availability of Se is so low that it causes very low uptake in plants which can ultimately lead to deficiency problems in animals. This is the first case series to report clinical WMD in foals in areas deficient in Se, in the Netherlands.

The aim of the current report is to provide an overview of the clinical history, symptoms and (clinical) pathology of 8 newborn foals living at 4 different premises and suffering from WMD together with the effectiveness of Se and vitamin E (Vit E) supplementation in the affected foals, their dams and herd members. Hands on practical information is provided to apply a correct and effective Se supplementation management in horses and which pitfalls need to be avoided for a successful approach.

**Case presentation:**

Case features and history were mapped out for all foals. Se and Vit E status were assessed for the foals, their dams and herd members, at admission and after 3 months of Vit E/Se supplementation.

Common symptoms were muscle weakness, inability to rise, lethargy and inadequate suckle reflex together with increased serum muscle enzymes and low glutathione peroxidase (GSH-Px) and low to normal serum vit E levels. Necropsy revealed necrosis of skeletal muscles consistent with nutritional myopathy. Se status of the dams and herd members correlated well with the Se status of the foals. All surviving foals (*n* = 6) showed normal Vit E and GSH-Px levels after supplementation, likewise, all horses tested at premises 1, 3 and 4. However, dams and herd members in premises 2 showed no normalization. Horses of that premises were diagnosed with pyrrolizidine intoxication one year prior to the study.

**Conclusions:**

Certain regions in the Netherlands are sufficiently Se deficient to predispose newborn foals to develop WMD, especially when they are being fed a diet that mainly consists of locally harvested roughage.

## Background

White muscle disease (WMD) is a peracute to subacute nutritional myopathy affecting both skeletal and cardiac muscles and is caused primarily by Se deficiency and to a lesser extend Vit E deficiency [1, 2]. It is reported in various animal species, including the horse, in which mainly foals are affected up to weanling age, though adult cases have been described occasionally as well, with 82% of confirmed cases aged below four years [3–12]. The underlying pathology is thought to be free-radical mediated rhabdomyolysis. Both Se and Vit E have an important antioxidant function and protect cell membranes against damage due to free radicals [13]. Se is an important, redox-sensitive element and is a constituent of GSH-Px, which is essential to neutralize reactive oxygen species produced during oxidation of unsaturated fatty acids. Se forms hydroxy fatty acids which are soluble and can be broken down by beta-oxidation. Vit E is important to prevent transition of fatty acids into peroxides, however, unlike Se, it can’t form soluble hydroxy fatty acids. Typically occurrence of WMD cases is geographically restricted to areas of low soil Se content. However not all foals low in serum Se and Vit E levels develop the disease, which suggests that individual factors (such as exercise load and other stressors) are also of importance in pathogenesis of the disease.

Mortality of WMD is high, both in foals and horses, ranging from 30% to 45% [5, 6, 14, 15]. Nutritional myodegeneration manifests as either a peracute form, where death due to cardiovascular collapse and pulmonary oedema occurs within hours; or more commonly, a subacute form where marked progressive muscular weakness and associated metabolic derangements (including hyperkalemia, hyponatremia, hypocalcemia, hypochloremia) generate a variety of clinical signs [6, 12], ranging from sudden recumbency, tachypnea, dyspnea and arrhythmias in the peracute form, to inability to stand, weakness, dysphagia, trismus and muscle fasciculations in the subacute form. In young foals these presenting signs must be differentiated from other potentially fatal conditions, such as septicaemia, neonatal asphyxia, pneumonia, botulism, tetanus, trauma or septic arthritis. Clinical pathology findings are consistent with a myopathy and the diagnosis of a nutritional myopathy can be supported by low serum gluthatione-peroxidase (GSH-Px) and Vit E levels [5, 6, 16]. At necropsy, affected muscle groups appear pale or exhibit pale striations. Since muscular exercise results in an increased production of free radicals and other forms of reactive oxygen species, muscles with high physiological motion activity such as the diaphragm, intercostal muscles, gluteal muscles, the tongue, masticatory muscles and the myocardium are usually most severely affected. These muscle groups typically have a high percentage of type 1 or red fibres. Histopathology reveals polyphasic and polyfocal degeneration and necrosis with hyalinization, fragmentation and mineralization of myofibres [17].

Se becomes more and more insufficient in food crops as a result of the increasing intensive plant production in many countries [18]. On top of that there is the problem of acid rain that reduces Se bioavailability. Effective recycling of selenium is challenging. Selenium is added in some commercial fertilizers, however, only a small fraction is taken up by plants, leaving much of the remainder to be lost for future utilization [19]. Therefore, problems associated with Se deficiency manifest themselves with increasing frequency and this trend is expected to continue [18–21].

Adequate Vitamin E and Se intake in mares in gestation is known to prevent WMD in newborn foals [1, 6, 22]. Se uptake in the foal mainly occurs via the placenta during gestation, whereas Vitamin E uptake occurs after birth during uptake of the colostrum. So uptake of Vit E and Se greatly depends upon Se and Vit E status of the dam [1, 23, 24]. Therefore pastures and fields growing fodder crops should be balanced in soil nutrient content, with special attention for Se content, in order to guarantee appropriate Se uptake by the fetus.

Breeding farms and farms specialized in rearing of young foals localised in Se deficient areas that provide a diet mainly consisting of roughage to their horses should be extra cautious. Unbalanced liming and fertilization conditions may further predispose their horses to health problems associated with insufficient Se uptake. Allaway [25] has suggested that it is desirable to control the Se levels in food and feed crops to a range between 0.1–1 ppm. In her extensive review on Se deficiency and toxicity Fordyce [26] suggests that Se levels in feed crops should go above 0.1 mg kg^−1^ and should not exceed 3–5 mg kg^−1^.

Little is known about how exactly Se is taken up by the horse via the oral route. Studies have shown that dietary Se supplementation, both through organic (selenomethioinin, e.g. through Se-enriched yeasts) and inorganic (selenite: SeO_3_^2−^) supplements,elevates plasma or serum and muscle Se status [22, 23]. Adequate Vitamin E uptake by the mare and subsequent redistribution to the colostrum requires appropriate intestinal absorption and liver function [2, 27]. Hepatic function can be chronically reduced as a result of progressive liver cirrhosis, a common consequence of ragwort poisoning in the horse [28].

**Fig. 1:**
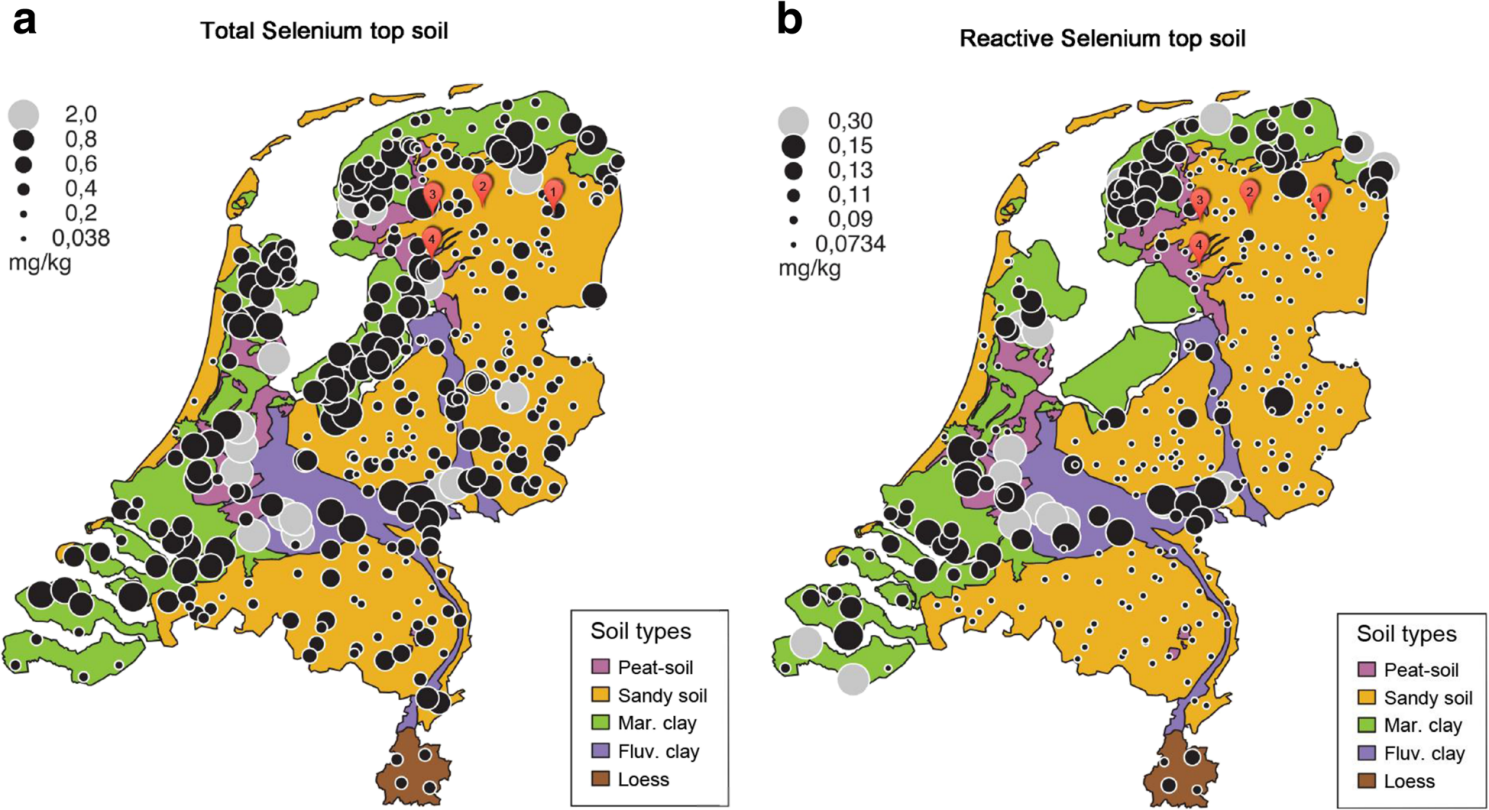
Geographical overview of Se content in the Netherlands. Notice that all four premises with affected foals are indeed located in Se deficient areas. Panel **a**: Results on top soil (0–20 cm) on 358 locations in forests, agricultural and natural areas, representing total Se content. Panel **b**: representing “maximum available Se content” (analysis with ICP-MS after 0.43 HNO_3_ extraction). Mark the important difference between “total” and “maximum available” Se content. The grey circles indicate the locations with Se concentrations exceeding the 95 percentile. Reprinted from Mol et al. [34]

**Fig. 2:**
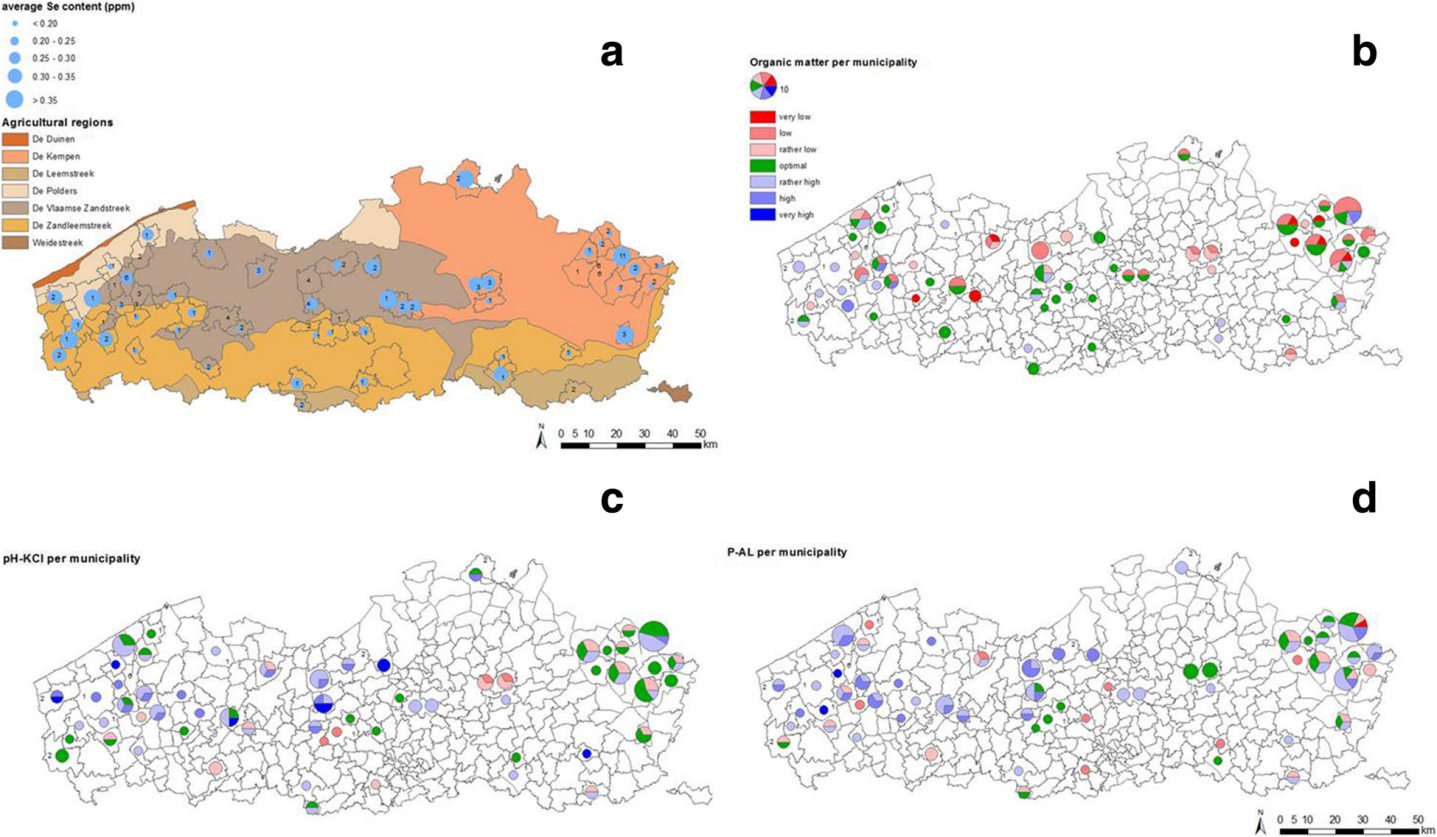
Geographical overview of Se content in top soil in Belgium (panel **a**) and top soil features of importance with respect to Se availability (panels **b**, **c** & **d**). Panel **a**: Geographical overview of Se content in Flanders. Results on top soil (0–6 cm) of 117 pastures between 2007 and 2015. Analyses with ICP-MS after HNO_3_-HCl (1:3) extraction. The number in the circles is the number of samples in the municipality. Results of the Soil Service of Belgium. Panels **b**, **c** & **d**: Geographical overview of soil fertility (0–6 cm) of 117 pastures between 2007 and 2015 in Flanders. Analyses of pH-KCl, O.M. with modified Walkley and Black, P-AL with ICP-MS after ammoniumlactaat extraction. Results of the Soil Service of Belgium. The size of the diagram corresponding with 10 samples per municipality is given in the legend

This is the first case series to report clinical WMD in foals in areas deficient in Se in the Netherlands and providing geological insight into possible preventive management measures. Effectiveness of Se and Vit E supplementation in these foals, their dams and herd members was monitored and deemed ineffective in adults of one herd formerly diagnosed with pyrollizidine intoxication based upon confirmed uptake of roughage contaminated with ragwort and increased blood liver enzymes. The soil content overviews are provided on geographical maps both for The Netherlands and Belgium (Figs. 1 & 2).

## Case presentation

Eight newborn foals living at 4 different premises (premises 1: cases 1 to 3, including follow up of Se and Vit E status of the respective dams and 4 herd members; premises 2: cases 4 to 6, respective dams and 7 herd members; premises 3: case 7; premises 4: case 8). Case features, history and clinical findings in the foals are summarised in Table 1. Figure 1 provides a geographical overview of total (panel A) and available (panel B) Se soil content in The Netherlands, together with the location of the four premises included in this case series. Clinical pathology at the time of admission in the foals and their respective dams are presented in Table 2. Increased muscle enzymes (CPK, SGOT and LDH) were found in all cases tested. Treatment of the foals upon admission encompassed plasma transfusion, infusion with normal saline, 20 mg/kg q24 IV amikacin^1^, 25 mg/kg q12 IV ampicillin^2^, 600 μg/kg q24 PO meloxicam^3^, and 4 mg/kg q24 PO omeprazol^4^. Also, these foals were manually supported to enable nursing at the mare. The foals in cases 3 to 8 received an injection of 250 mg Vit E and 7.5 mg Se^5^ on the day of admission. All foals, dams and herd members were orally supplemented with 2 mg/kg Vit E and 5 μg/kg Se (as sodium selenite: Na_2_SeO_3_)^6^ during three consecutive months.

**Table 1:** Case details of the foals

	Case 1	Case 2	Case 3	Case 4	Case 5	Case 6	Case 7	Case 8
Year presented	2007	2009	2010	2010	2011	2012	2011	2011
Age of foal	1 day	12 h	3 h	1 week	36 h	12 h	1 day	1 h
Behaviour	Depressed	Dull, uncontrolled movements	Bright	Bright	Dull	Bright	Dull	Bright
Muscle weakness	Not noticed	Not noticed	None	Sternal recumb, unable to remain standing	Lateral recumb	Unable to rise	Unable to rise	Not noticed
Suckling	Reflex absent	Reflex absent	Reflex absent	Normal	Normal	Normal	Normal	Normal
Gestation	Normal length (reference: 320–360 days [55]), no adverse events (illness, premature milk production, …)							
Parturition	Normal	Normal, bleeding from umbilicus	Normal	Normal	Normal	Normal	Normal	Mare rectum prolapse, foal normal.

**Table 2:** Blood values of foals on or shortly after presentation in hospital and GSH-Px and Vit E values of their respective dams

		Case 1 2007	Case 2 2009	Case 3 2010	Case 4 2010	Case 5 2011	Case 6 2012	Case 7 2011	Case 8 2011	Reference values
Hematocrit (%)		43	52	42	27	35	.38	40	25	32–52
WBC (g/L)		11.7	18	7.3	11.1	0.4	4.4	6.8	10.6	5.5–12.5
Electrolytes		†	*	†	*	K > 9	*	†	*	°
Blood gasses		†	*	†	*	pH 7.2	*	†	*	°°
Urea (mmol/L)		12.7	12.1	6.2	11.5	†	4.4	2.9	3.5	0–9
Creatinine (μmol/L)		*	518	250	44	†	95	93	79	116–180
GOT (IU/L)		*	*	82	6000	5960	4340	17,500	9120	22–488
CPK (IU/L)		*	*	710	33,300	172,800	39,700	137,300	95,400	0–269
LDH (IU/L)		*	*	1128	1678	51,000	14,100	83,000	52,600	162–412
IgG (g/L)		*	*	*	<4	*	*	*	4–8	>8
GSH-Px (U/g Hb)		*	*	43	22	*	*	12	11	>120 ‡
Vit E (μmol/L)		*	*	8.7	2.0	*	6.4	6,2	6.1	§
MARES	GSH-Px (U/g Hb)	*	*	59	9	*	*	<10	16	>120 ‡
	Vit E (μmol/L)	*	*	6.3	3.8	*	4.0	6,4	8.8	§

* not tested

† all within reference values

° References (mEq/L): Na (132–146), K (2.4–4.7), Cl (99–109), Ca (1.4–1.7) [56] °° References: bicarbonate (20–28 mmol/L), pH (7.32–7.44) [56]

‡ reference values for the laboratory used in case 4: 120–300, reference values the laboratory used in case 5, 6 and 8: 120–500

§ reference values for the laboratory used in case 4: >7,4, reference values the laboratory used in case 5,6,7 and 8: > 4,0

### Outcome

Cases 2 and 5 did not survive. Case 2 gradually became lethargic and due to complete absence of a suckling reflex, the owner opted for euthanasia 2 days after admission. Case 5 died four hours after admission. All other foals survived. Their suckle reflex improved and they regained strength over a period of days. However, it took more than 3 weeks before foal 7 eventually was able to rise by itself.

### Post mortem findings

Post mortem findings were available for the two non-surviving cases (case 2 and 5) and histopathological examination of respectively the iliopsoas and semimembranosus muscle revealed findings consistent with WMD.

The case 2 foal was found to have its gastro-intestinal tract poorly filled with ingesta, and the left iliopsoas muscle was markedly pale. The lungs were hyperaemic with mild pulmonary oedema. In both the alveoli and bronchioles a protein-rich fluid containing fibrin, erythrocytes, PMK’s and skin scabs was found, suggestive of aspiration of amniotic fluid possibly due to intra-uterine asphyxia. The left major psoas muscle showed swelling of muscle fibres, loss of cross striation and fragmentation of many myofibres, focal mineralization of affected muscle fibres, karyorrhexis and karyolysis consistent with WMD.

**Fig. 3:**
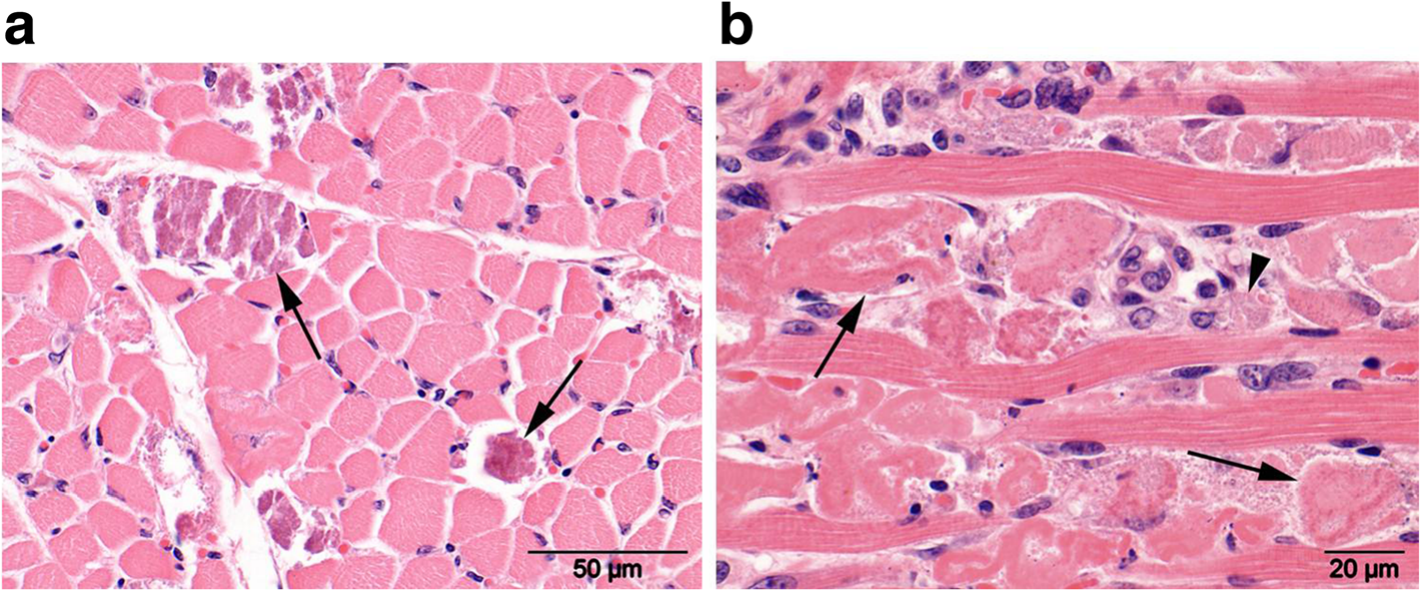
Histological image of a cross section of an affected skeletal muscle showing. Panel **a**: multifocal mineralization of muscle fibres (arrows). *H&E stain*. Panel **b**: Longitudinal section of a severely affected skeletal muscle revealing hyalinization and fragmentation of muscle fibres (arrows) and influx om macrophages phagocytizing myofibre remnants (arrowhead). *H&E stain*

Samples of heart muscle and semimembranosus muscle of case 5 were submitted for histopathological evaluation. Diffusely in the semimembranosus muscle the muscle fibres were hypereosinophilic, fragmented, vacuolated and showed loss of cross striation with influx of macrophages and hypertrophy of satellite cells. A small number of muscle fibres retained some striation and showed increased basophilia and centrally localised nuclei, suggestive of regeneration. The diffuse severe polyphasic degeneration and necrosis of the muscle fibres is consistent with WMD (Figure 3).

### Follow up

GSH-Px and Vit E values for the foals of premises 1 and 2, their dams and herd members at the time of admission and after three months of oral supplementation are depicted in Tables 3 and 4. The results of the analysis of the locally sourced grass and hay fed to the horses in premises 2 is provided in Table 5. Blood GSH-Px values improved after three months of supplementation in premises 1, however, no improvement was recorded in premises 2. Table 4 shows the blood results of 10 mares at premises 2 after a prolonged period of vitamin E and Se supplementation. 7/10 and 6/10 mares showed Se and Vit E deficiency respectively despite prolonged supplementation. These horses had a previous history of liver disease due to ragwort (pyrrolizidine) poisoning.

**Table 3:** GSH-Px and Vit E values of cases 2 and 3, their respective dams and two other herd members before and after three months of supplementation of Vit E and Se at premises 1

	GSH-Px (U/g Hb)		Vitamin E (μmol/L)	
	Before supplementation	After 3 months supplementation	Before supplementation	After 3 months supplementation
Mare case 3	59	189	6.3	5.0
Foal case 3	43	141	8.7	6.5
Mare case 2	48	154	6.2	6.4
Horse X	65	145	4.2	5.9
Horse Y	94	183	6.8	**7.3**
Reference value	120–300 U/g Hb		>4 μmol/L

**Table 4:** Post-supplementation GSH-Px and Vit E values of horses at premises 2. Horses with low GSH-Px had previously suffered from ragwort poisoning

	GSH-Px (U/g Hb)	Vitamin E (μmol/L)
1	246	5.1
2	36	1.8
3	65	1.4
4	63	5.6
5	24	3.8
6	154	3.5
7	254	5.5
8	31	2.6
9	26	4.0
10	43	1.8
Reference value	120–300 U/g Hb	>4 μmol/L

**Table 5:** Mineral analysis of grass and hay premises 2

	Grass	Hay	Reference values
Co (μg/kg DM)	89	88	>100
Na (g/kg DM)	1.6	2.7	3.0–30
Mg (g/kg DM)	2	2.2	3.9–5.0
Se (μg/kg DM)	29	44	100–1000
Fe (mg/kg DM)	117	175	50–100
Ca (g/kg DM)	4.1	3.0	2–7
P (g/kg DM)	3	3.0	2–5
K (g/kg DM)	17	10	> 4.3
Cu (mg/kg DM)	8.5	7.8	7.0–10
Zn (mg/kg DM)	46	39	40–100
Mn (mg/kg DM)	223	348	> 40

The results of the analysis of the roughage (Table 5) indicate that the mares from premises 2 were fed a Se deficient diet.

## Discussion

This case series shows that WMD in foals is a relevant disease in the Netherlands and that more attention needs to be paid to soil Se status, and soil pH and organic matter content in any geographical region that is known to have Se deficient soil. Indeed, it is important that Se fertilization receives proper attention. In most soils, Se is present in very low concentrations, sometimes even below 0.2 mg/kg. Even when total concentrations in soil seem adequate, soil conditions may be such that the bio-availability of Se is so low that it causes very low uptake in plants which can ultimately lead to deficiency problems in animals. A problem which will manifest itself most probably with increasing prevalence in the future [18–21]. See Figs. 1 & 2 for an overview of the relation between soil types and Se availability in The Netherlands (Fig. 1) and Belgium (Figure 2) and note the important differences between total Se (Fig. 1, panel a) and maximum available Se (Fig. 1, panel b) content in the top soil.

**Fig. 4:**
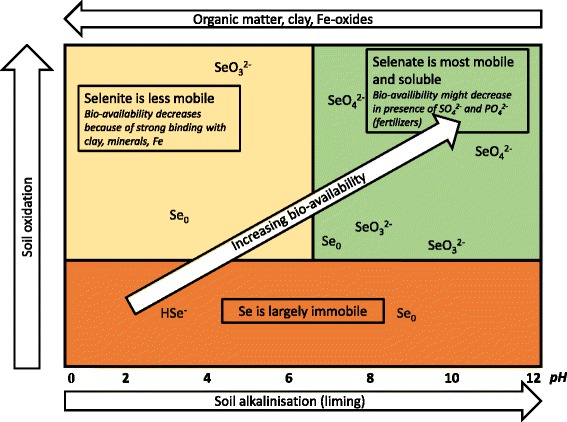
Schematic overview illustrating the influence of specific soil features such as pH on chemical behaviour and bio-availability of Se. Notice that the highly bio-available selenate is also most soluble and will therefore easily leach from well-drained soils. (Adapted from Fordyce [26])

Important to notice: in acid and neutral soils, Se availability is suppressed because Se is mostly present as selenite which may be fixed and highly insoluble as ferric selenite (Figure 4). Se can also form organic complexes that are generally not available to plants. Se may occur in soils in a number of oxidation states depending on soil redox potential, soil pH, microbiological effects and the presence of other ions like phosphate and sulfate, which are often added to fertilizers [25, 29, 30]. Selenate (SeO_4_^2−^), the ultimate oxidized form, which is also the form taken up by plants (high bio-availability), occurs only under well aerated alkaline conditions [26, 31]. However, selenate is also the most mobile form, therefore it tends to leach readily from well drained soils, that can become on their turn extremely low in Se content. When Se occurs in the less mobile selenite form, which is typical for acid or neutral soils, its availability is often hampered by absorption processes such as the binding of selenite to oxides, organic matter and clays or, precipitation as the insoluble ferric selenite in the presence of iron (Fe). Se can also form organic complexes that are generally not available to plants and that can leach from the soil. In very wet, chemical reduced soils like some peat and clay soils Se is usually present in insoluble, and thus unavailable, reduced forms (elemental or as selenides or sulfides). Recent research on Se speciation in 80 Dutch soil locations covering both grass land and arable land and covering all major soil types in the Netherlands, shows that most of the Se (80% on average) is present in organic forms whereas the inorganic form is mainly selenite [32, 33]. The overall low levels of Se in Dutch soils combined with the chemical speciation of Se can lead to very low bio-availability of Se in many areas in the Netherlands. And this seems to be a general trend, also in other countries.

The presence of sulfate (SO_4_^2−^) (present in certain fertilizers) inhibits the uptake of Se by plants and has a greater effect on the preferential selenate than on selenite. It was believed that a high content of phosphorus in the soil should increase Se uptake by plants as the phosphate (PO_4_^3−^) ion can readily be adsorbed in soils and can displace selenite from fixation sites. However, a recent study performed in the Netherlands, indicates that both SO_4_^2−^ and PO_4_^3−^ can also have a negative effect on Se uptake by plants [32]. A very complete overview of Se deficiency and toxicity in animals and the role of soil and plant Se content is provided by Fordyce et al. [26].

It is clear that many factors have their influence on Se bioavailability and thus proper management with respect to Se supplementation is quite challenging. Still, there are quite some tips that can help to support implementation of an effective Se supplementation management.

In horses, focus needs to be directed towards Se status of the dam, since the Se status of the neonatal foal depends on placental passage and thus the dams Se status during gestation. The dams milk is low in Se and is not considered as an important Se source for the foal [23]. This emphasizes the importance of soil analysis for grazing meadows, for pastures with roughage production and for parcels intended for fodder crop production as well as the analysis of roughage being fed to broodmares. Soils must be strategically managed in order to achieve an optimal fertility status for at least pH, organic matter and phosphate content, which in turn will stimulate appropriate Se-uptake by the crops. Figure 1 (panel a) shows the total top soil Se concentration in the Netherlands measured with ICP-MS after HF-destruction and for comparison, in panel B the potentially reactive (“available”) Se concentration in top soil (0–20 cm) samples is depicted [34]. Comparison of the two maps shows clearly that only part of the Se present in Dutch soils is actually available for uptake by plants, and that the maximum available levels in a large part of the country are too low for healthy Se levels in crops. A similar trend is seen in Belgium, as depicted in Fig. 2. Panel a, shows the map with the acid extractable contents of Se in the top soil (0–6 cm) of 117 pastures in Flanders (Belgium) between 2007 and 2015, measured with ICP-MS after extraction with HNO_3_-HCl (1:3). Both Figures clearly demonstrate that both in Flanders as well as in the Netherlands the Se content of the upper soil ranges in most cases around the background values of 0.2 to 0.5 ppm, as was also determined by De Temmerman et al. [35].

When Se-content in soil is lower than 0.6 ppm there is a clear risk for having deficient Se content in the grass or roughage produced on these soils and an inadequate Se uptake by livestock living and being fed fodder crops harvested on these grounds. A balanced soil fertility status stimulates the Se-uptake by the crop, and to reach this balanced fertility status, a near-neutral pH as well as a high phosphate content of the soil are beneficial. Figure 2, panels a, b and c show the soil fertility status of 117 pastures analysed by the Soil Service of Belgium on demand of the Flemish farmers. The soil fertility condition is classified in 7 classes: very low, low, rather low, normal (target value), rather high, high and very high. In most pastures the pH is within the target value or even more alkaline which increases the Se-availability together with the rather low organic matter content. About 16.5% of the pastures has a phosphate content within the target value and 58.8% above the target value, which stimulates the Se-uptake by the crop. Still, several regions are low in Se content (panel A). In cases where soil analysis reveals a Se-content <0.6 ppm, Se fertilization is recommended, together with correction of soil pH if needed. For grassland a fertilization of 2–4 g Se/ha is recommended for every grass cut with a maximum of 10 g Se/ha/year [36], a level of fertilization found in many countries, including the Netherlands [37]. The effect of Se fertilization can be evaluated by follow-up analysis of the Se content in the grass or roughage, since Se can potentially be toxic in case of excessive supplementation, either through the crops or as a result of leaching to ground and surface waters (because the mobile selenate form is mostly used for supplementation). In Finland and some other countries Se fertilization proved effective in increasing the Se levels in crops, animals and humans [38].

Possible interventions at soil management level mainly involve the choice of fertilizer. Some rock phosphate fertilizers are rich in Se, and in most countries where Se is permitted as a supplement to fertilizers, farmers can buy all kinds of fertilizers with added Se. Once, Se fertilization is applied, proper follow-up of the resulting Se soil and crop content should be performed to check whether additional oral supplementation of livestock is necessary. Oral supplementation can be achieved by applying top dressings on the feed, providing concentrates fortified with Se or by providing the animals with Se-enriched lick stones (although the latter approach entails thatthe uptake will greatly depend on the individual licking preference of the horse). Both inorganic Se (selenite) and organic Se (Se-enriched yeasts) supplements can be used to increase the Se status [39]. However, organic Se seems to have a higher digestibility [40] and be more effective to elevate total plasma Se because of higher selenomethionine levels [41]. Proteins incorporated with selenomethionine might serve as future Se reserve and explain why GSH-Px activity remains increased for a longer period after withdrawal of an organic Se supplement when compared to an inorganic Se supplement [42].

As mentioned previously, problems associated with Se deficiency are expected to occur with increasing prevalence. In the current presented case series, the most consistent clinical symptoms were muscular weakness, inadequate suckle reflexe and stillbirth, as reported in literature [6]. Clinical biochemistry showed severely increased serum muscle enzymes and low GSH-pX values. No direct measurement of blood Se levels was performed, however several authors have confirmed the strong correlation between blood Se and GSH-pX levels in both foals and horses [23, 43, 44]. The differential diagnosis for elevated muscle enzymes in foals includes in utero hypoxia (e.g. due to Equine Herpes Virus, placentitis), dystocia (peripartum asphyxia and muscle trauma) and hereditary myopathies (typical but not limited to Quarter Horses and related breeds) [45–47]. Both in utero hypoxia and dystocia are typically not associated with the extreme increases in muscle enzyme levels encountered in the current case series (>100-fold increase above reference range, except for case 3), in association with low GSH-Px levels [46, 48]. Regarding the hereditary myopathies, PSSM (polysaccharide storage myopathy) is typical for adult horses after the onset of training [45]. To our knowledge, the youngest foal described with CPK elevations due to PSSM was 1 month old and did not have any clinical signs yet [49]. GBED (glycogen branching enzyme deficiency) is fatal in all cases within the first two months after birth. Post-mortem macroscopic and histological signs are limited without the use of PAS staining, which is not in accordance to our findings in the two non-surviving cases [45].

Important to notice is that both in this study and in others, Se deficiency is more consistently present in WMD cases than is Vit E deficiency [1, 9]. Indeed, in our study, the Vit E status in all foals but one with WMD was normal. This was also true for the respective mares in which Vit E and GSH-pX was determined. It may be that Se deficiency is more likely to trigger muscular necrosis, whereas Vit E deficiency may be more related to fat necrosis [50].

Finally, an interesting finding in the current study was the fact that proper Se supplementation was ineffective to solve problems at one premises formerly diagnosed with ragworth poisoning. Here, foals suffering from WMD were still being born despite supplementing the dams with Vit E and Se for a prolonged period. All these post-supplementation WMD affected foals were born out of mares that one year before, had been diagnosed with ragwort poisoning. They still showed an insufficient Se status despite supplementation, probably due to a diminished liver function, although no disturbed blood liver enzymes were present anymore. The findings in our case series could warrant for a more thorough follow-up of Se status when supplementing Se to Se deficient horses suspected of having a reduced liver function. Although up until now, no equine study has investigated a possible link between reduced hepatic function (due to ragwort poisoning) and birth of Se depleted foals, a link between Se deficiency and liver disease is well-known in human medicine [51, 52]. Recently, Burk et al. [53] have demonstrated that in Se-deficient human patients with liver cirrhosis the selenomethionine metabolism is impaired and therefore organic Se (selenomethionine) supplementation is not effective, in contrast to inorganic Se (selenate) which does increase GSH-Px activity. However, in the current case series inorganic selenium (selenite) was supplemented and this could not increase GSH-Px activity either. It would be interesting to check in the future whether increased doses of inorganic selenium or perhaps another form of inorganic selenium (selenate instead of selenite) can offer a solution in such a situation.

## Conclusions

In order to prevent foals being born with White Muscle Disease in premises localized in areas with low Se soil content, routine soil Se content monitoring, together with analysis of the roughage fed to broodmares is recommended. As the uptake of Se by the crop is not only depending on the Se content of the soil but is also influenced by the general soil fertility condition especially soil pH, phosphate and organic matter content, a complete soil fertility analysis of the top soil is recommended every four years. Addtionally, monitoring Se status of broodmares and their herdmates (plama GSH-Px, serum/plasma/whole blood Se) is advisable in Se deficient regions. For the remediation of Se deficiency in animals various options are available, indirectly by improving the Se status of the soil by applying specialized fertilizers and directly by supplementing the intake of the animals through their feedstuffs. Structurally improving the Se status of the soil is generally the prefered choice in the long run [54]. Special consideration needs to be taken in broodmares suspected of having a reduced liver function, as the Se status may remain low despite oral Se supplementation.

### Manufacturers’ details

1. Amikacin®, Merial, Velserbroek, the Netherlands AST farma BV, Oudewater, the Netherlands
2. Ampi-Dry®, Dopharma, Raamsdonksveer, The Netherlands
3. Metacam®, Boehringer Ingelheim, Alkmaar, The Netherlands
4. Gastrogard®, Merck Schuchardt OHG, Hohenbrunn, Germany
5. Etosol®, Eurovet Animal Health BV, Bladel, the Netherlands
6. Professional’s Choice Supplements®, WD Holdings, London, United Kingdom

## Abbreviations

CPK: creatine phosphokinase
Fe: iron
GSH-Px: glutathione peroxidase
LDH: serum lactate dehydrogenase
Se: selenium
SGOT: serum glutamic oxaloacetic transaminase
Vit E: Vitamin E
WMD: white muscle disease
